# Expanding the Terpene Universe: Synthetic Biology and Non-Natural Chemistry in Engineered Microorganisms

**DOI:** 10.3390/molecules30204065

**Published:** 2025-10-13

**Authors:** Yueli Hu, Ziyan Yuan, Qian Wang, Ziyan Wang, Jianan Cao, Jiaxin Wu, Xinkun Ren

**Affiliations:** 1 College of Engineering and Applied Sciences, Nanjing University, Nanjing 210023, China; h18751850898@163.com (Y.H.); yuanziyan@nju.edu.cn (Z.Y.); 652024340036@smail.nju.edu.cn (Z.W.); cjnscience@163.com (J.C.); 2 School of Environment, Nanjing University, Nanjing 210023, China; wangqian@smail.nju.edu.cn

**Keywords:** terpenoid biosynthesis, metabolic engineering, microbial cell factory, synthetic biology, artificial metalloenzymes

## Abstract

Terpenes, representing one of the most extensive classes of natural products, hold significant value in the fields of pharmaceuticals, fragrances, and biofuels. Extracting these compounds from natural sources is often environmentally unsustainable, and the structural diversity found in nature is inherently limited. Metabolic engineering using microbial hosts offers a scalable and sustainable alternative, utilizing optimized biosynthetic pathways—such as the mevalonate (MVA) and the methylerythritol phosphate (MEP) pathways—to achieve high-yield production of natural terpene scaffolds. This review focuses on the various strategies in developing microbial cell factories, ranging from enhancing precursor supply to optimizing terpene synthase systems. A new and promising frontier is the increase in structural diversity of terpenes by integration of non-biological chemical transformations into engineered biosynthetic pathways. We discuss the use of artificial metalloenzymes such as engineered cytochrome P450 variants that catalyze non-natural carbene transfer reactions (cyclopropanation). The merging of synthetic biology and synthetic chemistry goes beyond the normal synthesizing capabilities found in nature, which may pave the way for the design of “non-natural” terpenoids that contain new additions and better capabilities.

## 1. Introduction

### 1.1. Terpenes: Biological Ubiquity and Industrial Significance

**Figure 1:**
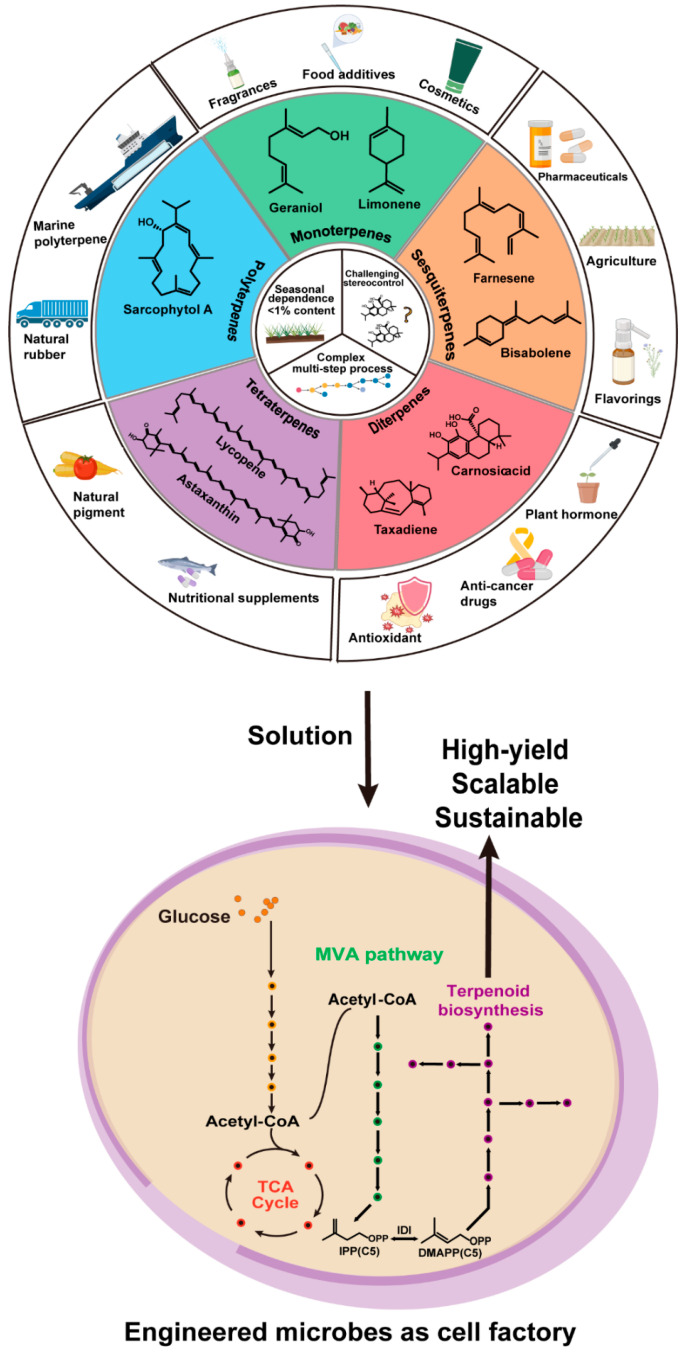
Overview of terpene structures, biosynthetic routes, and applications. (TCA, tricarboxylic acid cycle; MVA, mevalonate; IPP, isopentenyl diphosphate; DMAPP, dimethylallyl diphosphate).

Terpenes are the largest and structurally most diverse class of natural products; over 80,000 structures have been identified so far. In addition to their role as pigments, hormones, and antioxidants, terpenes are involved in various biological functions and mediators of ecological interactions [1,2,3,4,5]. They are as biologically important as they are industrially. Terpenes are essential components in both pharmaceutical and nutraceutical contexts (e.g., the malarial drug artemisinin), as flavor and fragrance agents, and as an auspicious candidate for second-generation biofuels (Figure 1). However, conventional production methods based on extraction from native plants or microbial sources tend to result in low yields, seasonal variations, and complex purification procedures, which render them economically and environmentally unviable for meeting global demand. While chemical synthesis is a powerful alternative, it is often impractical for complex terpene structures due to poor step economy and stereochemical control difficulty [6,7,8,9]. As a result, there is an urgent need to create strong, large-scale, sustainable production platforms.

Metabolic engineering of microbes appears to be the most effective solution to this problem. By modifying the central metabolism of genetically manipulatable organisms such as *Escherichia coli* and *Saccharomyces cerevisiae*, effective “cell factories” can be designed for the sustainable and scalable production of terpenes with high yield, as opposed to traditional extraction or synthetic methodologies (Figure 1) [10].

### 1.2. Expanding Beyond Nature’s Biosynthetic Toolkit

**Figure 2:**
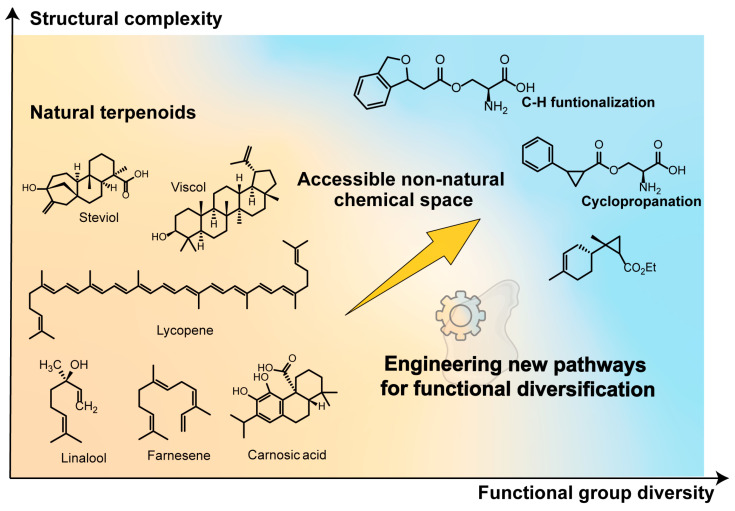
Conceptual expansion of the terpene chemical space. Natural terpenoids (**left**) occupy a limited region of structural complexity and functional group diversity. By integrating synthetic biology with abiotic transformations, new chemical modifications such as C–H functionalization and cyclopropanation (**right**) become accessible, enabling the design of non-natural terpenoids beyond the scope of nature.

Although microbial engineering has overcome the scalability challenges associated with producing many natural terpenes, a central limitation remains. The biosynthetic toolkit available in nature is large but ultimately limited. The naturally occurring terpenes show structural diversity that represents only a small fraction of the isoprenoid-derived chemical space theoretically accessible [11]. There are a whole bunch of terpenes that do not exist in nature, meaning it is a molecule that no living organism produces. More configurations are possible for the hollow fibers of polysaccharides and proteins than previously thought. Thus, these scaffolds may possess better properties such as improved bioactivity, better metabolic stability, or novel functions not present in nature. Despite significant advances in protein engineering and bioinformatics that have improved enzymatic activity and substrate selectivity, the natural diversity of enzymes is still largely confined to a limited set of chemical transformations, including cyclization, oxidation, and acetylation—reactions that are markedly narrower in scope compared to those routinely employed in modern synthetic chemistry. This limitation strongly motivates a shift from imitating natural biosynthetic pathways to engineering a larger “terpenome” with desirable properties, thus enhancing the potential of biosynthetic pathways (Figure 2).

### 1.3. Toward Programmable Biosynthesis of Terpenes

The review summarizes the transition from a “business as usual” approach to a “programmed” biosynthetic strategy to produce terpenes. To begin with, we first look at fundamental metabolic engineering approaches to addressing bottlenecks in production by increasing carbon flux through the mevalonate (MVA) and methylerythritol phosphate (MEP) pathways, engineering prenyltransferases to regulate precursors accurately, and harnessing terpene synthases to generate structural diversity in microorganisms [12]. Yet, this review contends that the future of the field lies in the integration of synthetic biology and synthetic chemistry. We look at how this approach solves the last barrier of natural diversity by incorporating non-biological catalytic functions into engineered living systems. By using metalloenzymes and catalytic systems, the hybrid strategy allows the functionalization of terpenes in vivo with non-natural chemical groups. With the powerful integration presented in this review, we have ushered in an era where biosynthetic systems need not be restrained to mimic nature but are instead empowered to surpass it for the programmable biosynthesis of specifically engineered terpenes with unique structure and function [13,14,15,16].

## 2. Foundation: Native Terpene Biosynthetic Pathways in an Engineering Context

The biosynthetic mechanism that produces terpenes is surprisingly conserved but produces structures that are very different from one another. For metabolic engineers, understanding this native blueprint is not simply a worthy academic pursuit, but a requirement for the successful redesign and optimization of biosynthetic systems. Here we have outlined the major pathways that have the essential parts. In particular, we will focus on key decision points, metabolic bottlenecks, and modular elements that can facilitate the engineering of microbial production platforms.

### 2.1. The Two Source Pathways: MVA vs. MEP—An Engineering Perspective

**Figure 3:**
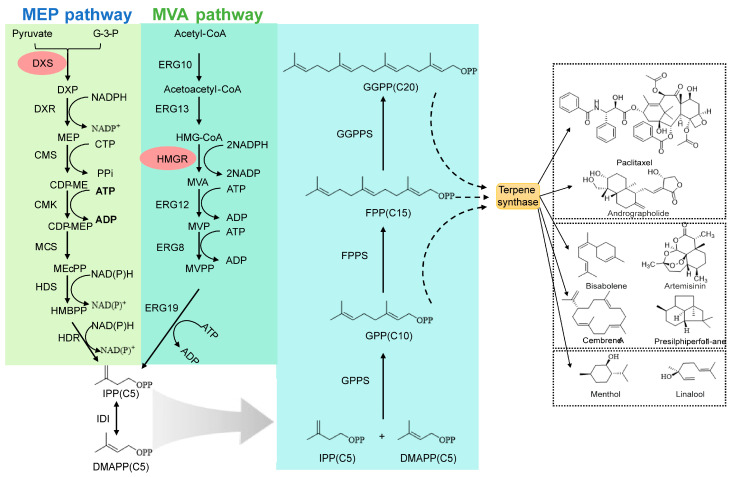
The framework of terpene biosynthesis: An engineering overview. (MVA, mevalonate; MEP, methylerythritol phosphate; IPP, isopentenyl diphosphate; DMAPP, dimethylallyl diphosphate; DXS, 1-deoxy-d-xylulose-5-phosphate synthase; DXP, 1-deoxy-d-xylulose-5-phosphate; DXR, DXP reductoisomerase; CMS, CDP-ME synthase; CDP-ME, 4-(cytidine 5′-diphospho)-2-C-methyl-d-erythritol; CMK, CDP-ME kinase; CDP-MEP, 2-phospho-4-(cytidine 5′-diphospho)-2-C-methyl-d-erythritol; MCS, MEcPP synthase; MEcPP, 2-C-methyl-derythritol-2,4-cyclodiphosphate; HDS, 4-hydroxy-3-methylbut-2-enyl-diphosphate synthase; HMGR, 3-hydroxy-3-methylglutaryl-CoA reductase; HDR, 4-hydroxy-3-methylbut-2enyl diphosphate reductase; ERG10, acetoacetyl-CoA thiolase; ERG13, hydroxymethylglutaryl-CoA synthase; ERG12, mevalonate kinase; ERG8, phosphomevalonate kinase; ERG19, mevalonate diphosphate decarboxylase; IDI, isopentenyl diphosphate isomerase; MVP, mevalonate-5-phosphate; MVPP, mevalonate-5-diphosphate; G-3-P, Glyceraldehyde 3-phosphate; HMBPP, 1-hydroxy-2-methyl-2-(E)-butenyl 4-diphosphate; HMG-CoA, 3-hydroxy-3-methylglutaryl-CoA; GPPS, geranyl diphosphate synthase; FPPS, farnesyl diphosphate synthase; GGPPS, geranylgeranyl diphosphate synthase, GPP, geranyl pyrophosphate; FPP, farnesyl pyrophosphate; GGPP, geranylgeranyl pyrophosphate; IPP, isopentenyl pyrophosphate).

All types of terpenes are formed from five-carbon structures called isoprenes, which are referred to as isopentenyl diphosphate (IPP) and its isomer dimethylallyl diphosphate (DMAPP). The mevalonate (MVA) pathway and the methylerythritol phosphate (MEP) pathway are two evolutionarily distinct metabolic routes responsible for the catalysis of these precursors [17,18,19,20,21] (Figure 3). When deciding on a pathway, it is important to note how much it affects the host organism and the strategies that it designs for metabolic engineering (Table 1).

The MVA pathway is a eukaryotic native pathway that forms the precursors for the biosynthesis of isoprenoid lipids by yeast *S. cerevisiae*, archaea, and the cytosol of plants through six reactions, starting from the two-carbon building block acetyl-CoA [22,23,24,25,26]. From a metabolic engineering perspective, a key advantage is localization in the cytosol, easing genetic manipulations that might suffer the hindrance of subcellular compartmentalization (Figure 3). One difficulty that metabolic engineers face is the bottlenecking of the pathway by the 3-hydroxy-3-methylglutaryl-CoA reductase (HMGR) [27,28,29]. The presence of the enzyme is strictly regulated and has a high metabolic cost, using two NADPHs per IPP. To solve this problem, engineering strategies often make use of a truncated and deregulated HMGR variant.

The MEP pathway originally exists in most bacterial species, including the widely used *E. coli*, and works in the plastids of plants [30]. The first reaction is condensation of G3P and pyruvate. From this condensation, a seven-enzyme reaction produces isopentenyl diphosphate, DMAP with the help of these reactions (Figure 3). The MEP pathway is commonly seen as more efficient than the MVA pathway because it has a higher theoretical carbon yield and consumes less ATP [31]. One primary engineering constraint lies in the first committed step, which is catalyzed by 1-deoxy-d-xylulose-5-phosphate synthase (DXS), which has low catalytic efficiency. The next step, catalyzed by DXS reductoisomerase (DXR) and inhibited by the antibiotic fosmidomycin, is sometimes exploited to select or screen for mutants.

**Table 1:** Engineering considerations for the MVA and MEP pathways.

Product	Host	Pathway	Bottleneck	Engineering Strategy	Maximum Titer
Sclareol	*Y. lipolytica*	MVA	Flux imbalance; unsuitable chassis; low enzyme activity	Enzyme engineering; increasing GGPPS supply	12.9 g/L [32]
Amorpha-4,11-diene	*E. coli*	MEP	Low growth-coupled production	Semi-continuous biomanufacturing	8.32 g/L [33]
Artemisinic acid	*S. cerevisiae*	MVA	Low expression of enzyme	Plant dehydrogenase introduction; additional cytochrome	25 g/L [34]
Bisabolene	*S. cerevisiae*	MVA	Growth limitations; insufficient precursors	MVA pathway enhancement; temperature-sensitive regulation	18.6 g/L [35]
β-Farnesene	*Y. lipolytica*	MVA	Insufficient precursors; flux imbalance	Acetyl-CoA boosting; large-scale optimization	35.2 g/L [36]
(*S*)-linalool	*Pantoea ananatis*	MVA	Poor enzyme compatibility; insufficient precursors	Increasing protein solubility; elevating precursor or supply; dual-phase fed-batch fermentation	10.9 g/L [37]
Geranylgeraniol	*Y. lipolytica*	MVA	Flux imbalance; several rate-limiting enzymes	Overexpressing bottleneck enzymes; expanding acetyl-CoA pool; downregulating FPP flux	3.3 g/L [38]

An important strategic issue deals with the choice of biosynthetic pathway when functioning with non-native hosts in metabolic engineering. Often, the native pathway of the chassis organism is either supplemented or even completely replaced. For example, the full MVA pathway is often introduced into *Escherichia coli* to enhance or bypass its endogenous MEP pathway, as the MVA pathway can provide higher metabolic flux when properly optimized [39,40].

### 2.2. Building Key Precursors: Prenyltransferases as Nodes for Flux Control

**Figure 4:**
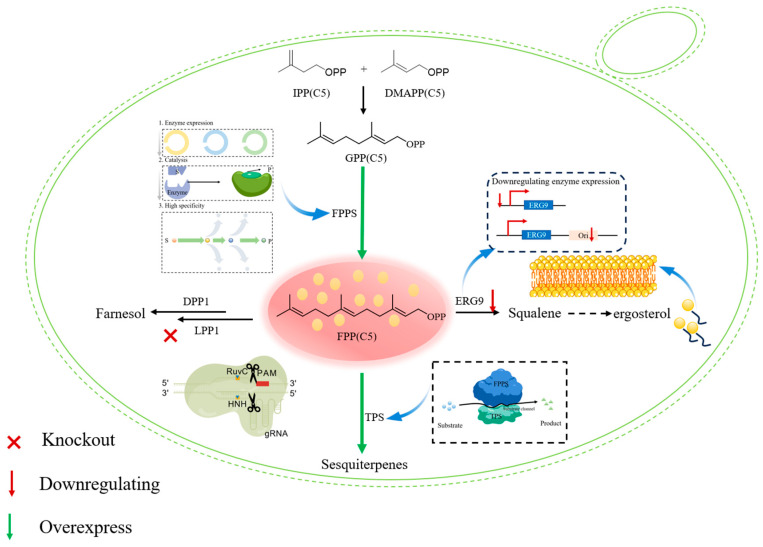
The prenyltransferase engineering challenge (PAM, protospacer adjacent motif; HNH, histidine–asparagine–histidine nuclease domain; IPP, isopentenyl diphosphate; DMAPP, dimethylallyl diphosphate; GPP, geranyl diphosphate; FPP, farnesyl diphosphate; FPPS, farnesyl diphosphate synthase; TPS, terpene synthase; DPP1, Diacylglycerol Pyrophosphate Phosphatase 1; LPP1, Lipid Phosphate Phosphatase 1; ERG9, Ergosterol Reductase).

IPP and DMAPP are condensed by prenyltransferases to generate the immediate precursors for all classes of terpenes. These enzymes represent key regulatory nodes for directing metabolic flux toward specific product classes (C10, C15, C20, etc.), making them central targets for metabolic engineering. Several engineering strategies have been developed to address the prenyltransferase challenge. These include the downregulation of ERG9 to decrease flux into sterol biosynthesis, CRISPR-based editing of competing pathways to minimize metabolic drain, and the introduction of heterologous prenyltransferases with tailored chain-length specificity. In addition, fusion proteins linking prenyltransferases to terpene synthases have been designed to promote substrate channeling and reduce byproduct formation (Figure 4). Geranyl diphosphate synthase (GPPS) catalyzes the condensation of IPP with DMAPP to produce geranyl diphosphate (GPP, C10), which serves as the precursor for monoterpenes [2,41,42,43,44]. Farnesyl diphosphate synthase (FPPS) catalyzes the addition of another IPP unit to GPP, forming farnesyl diphosphate (FPP, C15), the universal precursor of sesquiterpenes, sterols, and ubiquinone. Geranylgeranyl diphosphate synthase (GGPPS) further extends FPP by incorporating an additional IPP unit to yield geranylgeranyl diphosphate (GGPP, C20), the precursor for diterpenes and carotenoids.

The engineering challenge stems from the competition for these central metabolic precursors. In *E. coli*, farnesyl pyrophosphate (FPP) is a native metabolite essential for maintaining cell membrane integrity. Consequently, redirecting significant amounts of FPP toward the biosynthesis of heterologous sesquiterpenes can lead to cellular toxicity and trigger regulatory feedback mechanisms. To mitigate these challenges, several strategies have been developed: downregulating endogenous FPP-consuming pathways to reduce competitive flux; expressing heterologous prenyltransferases with high specificity for the desired product chain length to direct metabolic flux more efficiently; and utilizing fusion proteins that physically tether a prenyltransferase to a terpene synthase, thereby creating a substrate channeling system that restricts intermediate diffusion and reduces off-target reactions [45,46].

### 2.3. Generating Carbon Skeletons: Terpene Synthases as Modular Plug-And-Play Units

In the biosynthetic pathway, TPSs represent the essential transformative enzymes. The linear prenyl diphosphates (GPP, FPP, and GGPP) are acted upon by these enzymes, and a range of complex cyclization and rearrangement reactions takes place. These are major reactions and are responsible for the production of the respective carbon skeletons of all terpene classes. Researchers working on metabolic engineering view TPSs as modular, interchangeable elements suitable for inclusion in a standardized, high-flux microbial chassis system engineered to supply the required precursor [47,48,49,50,51,52,53].

The catalytic process typically starts with the ionization of the diphosphate group to create a highly reactive carbocation, followed by a series of cyclization reactions, hydride shifts, and Wagner–Meerwein rearrangements. The reaction eventually ends by deprotonation or nucleophilic capture (most commonly water), which dictates the olefinic or oxygen nature of the final product. From the point of view of metabolic engineering, several enzymatic properties of TPSs are important: (1) Product promiscuity: Many TPSs produce a mixture of a major and minor product from one substrate, making downstream purification more challenging. Scientists often use protein engineering methods to create “designer” TPSs with customized product specificity or improved catalytic fidelity. The plant-derived TPSs are heterologously expressed in microbial hosts, which often display misfolding, insolubility, and low enzyme activity. To tackle these challenges, functional expression levels of the proteins are often raised through codon optimization and use of fusion tags. New compounds can be generated by introducing a different TPS gene into the generic FPP- or GGPP-overproducing strain. This modularity at this stage of the pathway underlies many of the microbial screening platforms used in natural product discovery and development. Understanding the trade-off between mevalonate (MVA) and methylerythritol phosphate (MEP) pathways, the key role of prenyltransferases as nodes of metabolic flux control, and the modularity of terpene synthases can allow metabolic engineers to tailor microbial systems for the high-level biosynthesis of native and non-native terpenes systematically.

## 3. Metabolic Engineering Strategies for Microbial Terpene Production

### 3.1. Foundational Platform Design: Chassis Selection and Central Metabolism Optimization

The design of the microbial host for efficient microbial production of terpenoids starts with the design of the platform, which involves host selection and basic metabolic rewiring. Whether to go for *E. coli* or *S. cerevisiae* is a matter of great choice, as each of these offers distinctive advantages based on different cellular and metabolic characteristics. *E. coli* has great genetic tractability, fast growth rates, and a robust toolkit for molecular manipulation that aids in engineering. The native methylerythritol phosphate (MEP) pathway has theoretical advantages in terms of carbon efficiency. However, this comes at the cost of limitations in acetyl-CoA availability. There are also challenges in expressing complex eukaryotic enzymes. In contrast, *S. cerevisiae* has complementary advantages, such as an MVA pathway, strong acetyl-CoA generation capability, and a eukaryotic cell architecture that supports expression of membrane-associated enzymes [54]. Moreover, due to its GRAS status, it is well-suited for use in pharmaceuticals and nutraceuticals. However, there are engineering challenges arising from its slower growth and complex genetics.

The metabolic redirection refers to an important step in platform development that involves shifting carbon flux from in vivo metabolic pathways towards the production of terpene precursors. In systems using the MVA pathway, this is enhancement of availability of acetyl-CoA by using multiple complementary ways. These include overexpressing deregulated pyruvate dehydrogenase complexes, engineering acetate recycling pathways, and suppressing competing acetyl-CoA-consuming reactions. For platforms using MEP, optimization involves boosting pyruvate and glyceraldehyde-3-phosphate levels by enhancing glycolytic flux and carefully balancing other intermediates [43]. Advanced computational approaches are increasingly applied to identify unconventional knockout targets that improve precursor yield while retaining viability.

Cofactor management is another important aspect of platform design. Both of the main terpene biosynthesis pathways had major redox and energy demands. For example, the production of isopentenyl diphosphate (IPP) via the MVA pathway consumes multiple equivalents of NADPH per cycle. This limitation can be addressed by engineering pentose phosphate pathway flux or incorporating various NADPH regeneration systems. Furthermore, both pathways consume considerable quantities of ATP so that the energy charge of the cell is maintained by coordinating the growth and production phases. Innovative approaches involve the implementation of synthetic transhydrogenase systems for NADPH regeneration and dynamic regulation strategies for uncoupling energy demand during growth from production, leading to optimal cofactor availability during fermentation and production of the desired product. This foundation provides the platform for more targeted pathway engineering interventions discussed in the following section.

### 3.2. Pathway Engineering and Optimization Strategies

**Figure 5:**
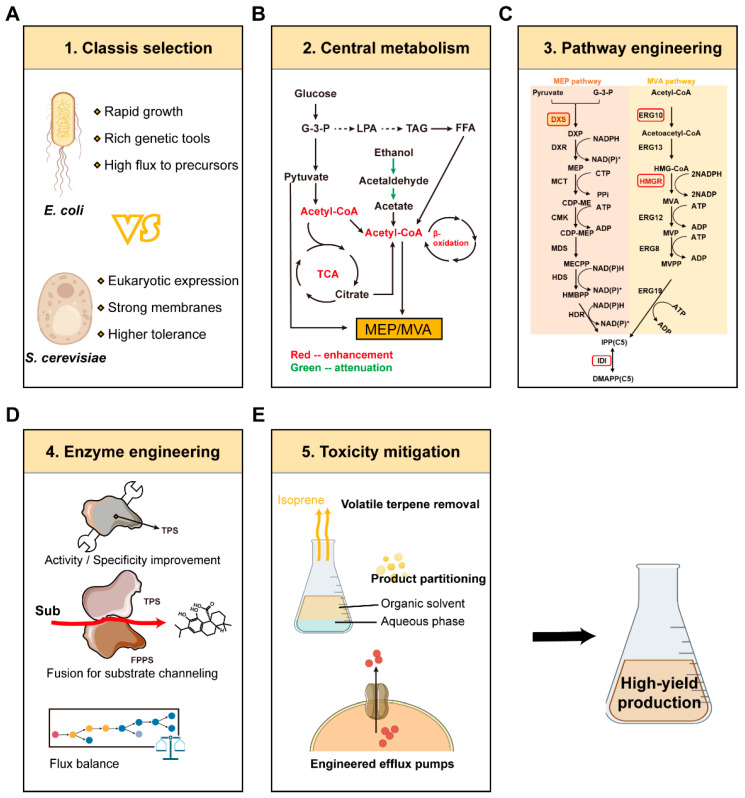
Comprehensive metabolic engineering workflow for microbial terpene production. (**A**) Chassis selection: Comparison of *E. coli* and *S. cerevisiae* as hosts. (**B**) Central metabolism optimization: Rerouting carbon flux to acetyl-CoA and balancing glycolysis and the TCA cycle. (**C**) Pathway engineering: modification of key MEP/MVA enzymes. (**D**) Enzyme engineering: Enhancing terpene synthase activity and specificity, including FPPS–TPS fusion constructs. (**E**) Toxicity mitigation: Strategies such as in situ product removal and efflux pump engineering (TCA, tricarboxylic acid cycle; MEP, 2-C-methyl-d-erythritol 4-phosphate pathway; MVA, mevalonate pathway; G-3-P, glyceraldehyde-3-phosphate; LPA, lysophosphatidic acid; TAG, triacylglycerol; FFA, free fatty acid; DXS, 1-deoxy-d-xylulose-5-phosphate synthase; MCT, 2-C-methyl-d-erythritol 4-phosphate cytidylyltransferase; CMK, 4-(cytidine 5′-diphospho)-2-C-methyl-d-erythritol kinase; MDS, 2-C-methyl-d-erythritol 2,4-cyclodiphosphate synthase; HDS, 4-hydroxy-3-methylbut-2-enyl diphosphate synthase; HDR, 4-hydroxy-3-methylbut-2-enyl diphosphate reductase; ERG12, mevalonate kinase; ERG8, phosphomevalonate kinase; ERG19, mevalonate diphosphate decarboxylase; ERG13, hydroxymethylglutaryl-CoA synthase; HMG1 (HMGR), hydroxymethylglutaryl-CoA reductase; IDI, isopentenyl diphosphate isomerase; IPP, isopentenyl pyrophosphate; DMAPP, dimethylallyl pyrophosphate; TPS, terpene synthase; FPPS, farnesyl pyrophosphate synthase; Sub, substrate; and β-oxidation, beta oxidation).

Pathway engineering focuses on eliminating intrinsic bottlenecks within terpenoid biosynthesis pathways through targeted enhancement of key enzymatic steps. In the MEP pathway, 1-deoxy-d-xylulose-5-phosphate synthase (DXS) is the primary bottleneck, and its overexpression often yields the best improvements. Enhancing isopentenyl diphosphate isomerase (IDI) ensures balanced IPP and DMAPP pools, which are crucial for downstream prenyltransferases. In the MVA pathway, 3-hydroxy-3-methylglutaryl-CoA reductase (HMGR) represents the major control point. The use of truncated, deregulated HMGR variants deprived of membrane-binding domains and feedback inhibition insensitivity is critical for achieving high metabolic flux through this pathway. Further improvements are possible through optimizing the subsequent steps (Figure 5).

Regulating competitive pathways is an important means by which to increase precursor availability. In *S. cerevisiae*, to enhance the production of sesquiterpenes, the suppression of the squalene synthase enzyme (ERG9) is needed, as it has been shown that the majority of farnesyl diphosphate will be pulled toward the sterol biosynthetic pathway [55]. This is typically performed through the realization of a promoter replacement in a tunable or repressible expression system, allowing the required balance of essential sterol synthesis and terpene production. The ispDF operon in *E. coli* utilizes isopentenyl pyrophosphate for cell wall biosynthesis. This is a major competitive drain. CRISPR interference or promoter engineering significantly boosts precursor/scaffold availability in its downregulation. Nonetheless, the complexity of the relation of such an intervention with the cellular fitness needs to be studied in detail. In an event where complete disruption of an essential native pathway is performed, expert choices of nutrient supplementation or dynamic regulatory strategies are required. In the absence of the above, there will be a risk of compromising cellular viability at the expense of maximum terpene yield.

Prenyl diphosphate pool management is also critical [56]. Farnesyl diphosphate and related intermediates are cytotoxic at high concentrations and vulnerable to competing reactions. One solution is substrate channeling via fusion proteins that tether prenyltransferases and terpene synthases, restricting intermediate diffusion and reducing off-target reactions. Fine-tuning expression levels—by testing different promoter strengths or ribosome binding site sequences—further optimizes flux, maximizing terpene formation while preventing intermediate accumulation.

Engineering terpene synthases further improves functional expression and catalytic performance in heterologous hosts. Plant terpene synthases often show poor solubility and aggregate when expressed in microbes. Solubility-enhancing fusion tags and codon optimization are, therefore, necessary. Protein engineering techniques, including directed evolution (mutant libraries and high-throughput screening) and rational design (structure-guided active site modification), have been applied to improve product specificity, catalytic rates, and stability [57]. Nevertheless, system-level considerations remain essential to balance flux improvements with host viability, as discussed in Section 3.3.

### 3.3. Advanced System Integration and Toxicity Mitigation

**Figure 6:**
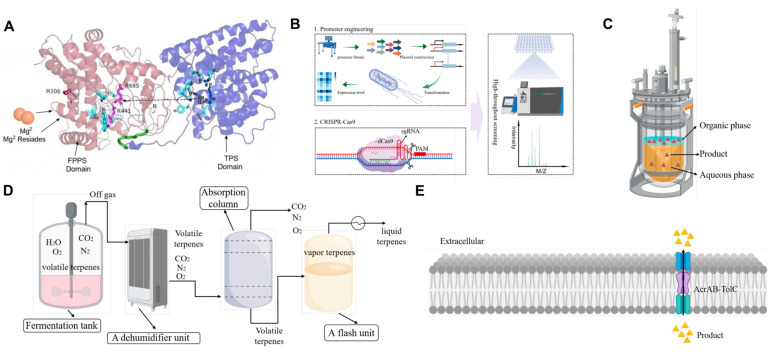
Strategies for alleviating metabolic competition and product toxicity in microbial terpene production: (**A**) substrate channeling via a fusion protein between farnesyl diphosphate synthase (FPPS) and a terpene synthase (TPS); (**B**) regulation of competitive pathways through promoter engineering or CRISPR interference (CRISPRi); (**C**) two-phase fermentation for in situ product removal; (**D**) gas stripping apparatus for volatile terpene recovery; (**E**) enhanced product export via native efflux pumps (FPPS, farnesyl pyrophosphate synthase; TPS, terpene synthase; sgRNA, single-guide RNA; PAM, protospacer adjacent motif; M/Z, mass-to-charge ratio; TolC, AcrA, and AcrB, components of the AcrAB–TolC efflux pump complex).

Beyond individual pathways, advanced system integration tackles the broader conflict between terpene production and microbial viability. Many terpenes are inherently cytotoxic, disrupting membrane integrity and inhibiting cell growth. To address this, product removal strategies are widely applied. Two-phase fermentation systems that use dioctyl phthalate or oleyl alcohol and other water-immiscible organic solvents can segregate terpenes based on their LogP values. They also prevent the concentration in the aqueous phase from reaching inhibitory levels. In the absence of two-phase systems, high concentrations of terpenes in fermentations produce toxic effects that hinder downstream recovery (Figure 6). Gas stripping achieves greater efficiency with volatiles through sparging (with air or inert gas) to send volatiles to condensation systems. Nevertheless, the added energy cost for the process makes it less effective for low volatility compounds. Beyond direct toxicity alleviation, dynamic regulation strategies have also been developed to balance growth and production phases, further enhancing productivity [58].

Dynamic regulation systems have emerged as the newest metabolic engineering tool that allows for temporal control over pathway expression for terpenes. By using metabolic valves and conditional expression systems to decouple growth and production, the redirection of the metabolism to terpenes happens only after the maximum biomass accumulation. Some examples are temperature-inducible promoters, nutrient-responsive regulatory components, and quorum-sensing-based systems that activate production pathways at specific cell densities. By avoiding the fitness burden of having the pathways on during the growth phase, and maximizing production during the stationary phase, these strategies can drastically enhance productivity and final titer.

Scale-up considerations address the unique challenges of translating laboratory-scale achievements into industrial production, including limitations in oxygen transfer, formation of nutrient gradients, and shear stress effects on engineered strains. To effectively scale up any bioprocess, it is important to integrate the bioprocess parameters with metabolic engineering parameters. For this, we need to consider the mixing time, mass transfer coefficients, heat generation at large volumes, etc. In addition, at commercial scale, substrate cost, downstream processing, and volumetric productivity become very important. Hence, these should be integrated into metabolic engineering design at an early stage. The cases that have had the most success with microbial terpenes see the early integration of strain engineering and bioprocess development so that lab developments can be easily translated to industry.

Collectively, these strategies optimize microbial robustness and production yield. The following section (Section 4) shifts focus from improving scaffold titers to broadening structural diversity, examining natural and abiological enzymatic toolkits for terpene diversification.

## 4. Expanding the Enzymatic Toolkit: Diversification Reactions

### 4.1. Natural Modifying Enzymes and Their Functional Expression

The primary mechanism for terpene diversification in nature involves specialized modifying enzymes that catalyze the functionalization of core carbon skeletons. Among these, cytochrome P450 monooxygenases are the largest class of oxygenases, capable of selective introduction of oxygen in regioselective and stereoselective hydroxylation. In vivo, these hemoproteins require electron transfer partners, most commonly NADPH-cytochrome P450 reductases (CPRs). The functional expression of plant P450s in microbes is hindered by their membrane dependence, folding complexity, and strict CPR isoform specificity.

Other modifying enzymes include dehydrogenases (redox transformations), methyltransferases (methyl group transfer), acyltransferases (acyl moiety attachment), and glycosyltransferases (sugar conjugation). Each class presents unique expression challenges, requiring codon optimization, coexpression with chaperones, or membrane engineering for correct localization and activity.

P450 expression is particularly problematic due to low coupling efficiency, in which NADPH is consumed without productive substrate turnover. This metabolic burden reduces pathway efficiency and can generate reactive oxygen species. Despite these challenges, landmark successes such as artemisinin biosynthesis in engineered yeast have demonstrated that extensive protein engineering and host adaptation can overcome barriers.

### 4.2. The Engineering Challenge of Natural Diversification

**Figure 7:**
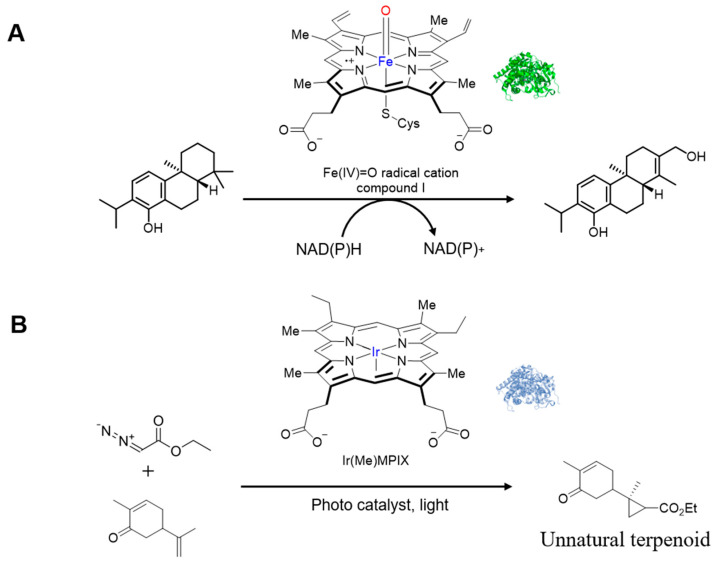
Comparative diagrams of natural P450 catalysis versus artificial metalloenzyme catalysis. (**A**) Natural hydroxylation reaction type catalyzed by cytochrome P450 enzyme; (**B**) Unnatural cyclopropanation reaction catalyzed by iridium-based artificial cytochrome P450 enzyme. The blue “Fe” and “Ir” indicate the iron and iridium atoms at the metal centers of the natural and artificial metalloenzymes, respectively, while the red “O” represents the oxygen atom in the Fe(IV)=O intermediate.

It is difficult to execute natural diversification strategies in engineered microbial systems for various fundamental reasons beyond functional expression. The complex structure and stringent cofactor requirements of the cytochrome P450 system are a good example of these complexities. Because it relies on NADPH as the principal electron donor, it creates a substantial metabolic strain as two moles of this reduced cofactor are consumed per cycle of catalysis. In microbial systems where terpenoid-based pathways already demand considerable amounts of NADPH, this extra utilization may severely curb the overall productivity of the pathway. Also, the redox reaction between P450 and its redox partner CPR must be optimized well enough for efficient electron transfer but not too well that it sets off a chain of uncoupled reactions leading to reactive oxygen species (Figure 7).

A major limitation of P450s is coupling efficiency, which is often below 50% in heterologous systems [59,60,61]. This wastes reducing power and stresses cells through oxidant formation. Their membrane-bound nature further complicates subcellular localization and integration. Finally, most native P450s show narrow substrate specificity, restricting their utility for generating diverse derivatives. These constraints have motivated exploration of abiological diversification strategies that expand chemical space beyond the limits of natural enzyme systems.

### 4.3. Introducing Abiological Chemistry: Expanding Nature’s Repertoire

**Figure 8:**
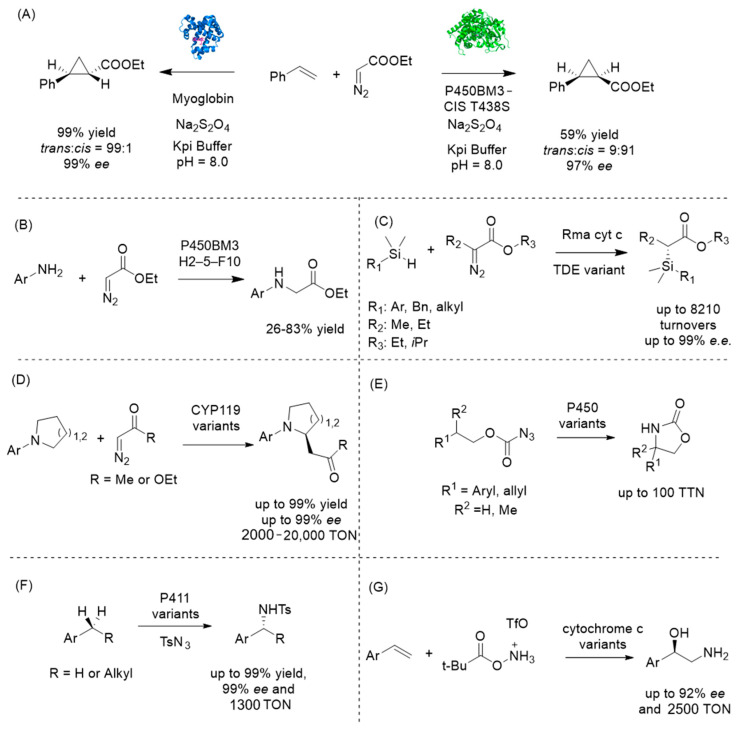
Representative examples of abiological P450 carbene and nitrene transfer catalysis: (**A**) cyclopropanation; (**B**) carbene N–H insertion; (**C**) carbene Si–H insertion; (**D**) carbene C–H insertion; (**E**) intramolecular nitrene C–H amination; (**F**) intermolecular nitrene C–H amination; (**G**) hydroamination.

Owing to the limitations of the natural diversification systems, several innovative strategies have been devised by incorporating abiological chemistry into biology. This conceptual advance transcends the capacity of nature’s enzymes to access transformations that cannot be achieved by living systems. In this new field, Hartwig, Keasling, and their research groups have successfully shown that synthetic carbene transfer chemistry can work in living microbes for additional terpene functionalization. This strategy is particularly designed to access a new chemical space that will not be possible through traditional enzymes, to create carbon-carbon bonds through carbene insertion reactions. In the seminal work on biocatalytic carbene and nitrene transfer chemistry by Arnold and Fasan [62,63,64,65,66,67,68,69,70,71,72,73,74,75,76,77,78], cytochrome P450BM3 and myoglobin were engineered to facilitate a variety of novel transformations (Figure 8) that were not found in nature, and a replacement of cysteine ligand with serine could significantly improve the reactivity, suggesting the importance of electronic properties of haem.

Building upon these observations, Keasling and Hartwig et al. modified the haem coordination environment by utilizing the iridium-based artificial cofactor Ir(Me)MPIX (MPIX for mesoporphyrin IX) [79,80,81,82]. The engineered artificial variant demonstrated a similar trend in regioselectivity and stereoselectivity as its native counterpart. This artificial metalloenzyme is able to insert carbene groups into olefinic linkages in terpene scaffolds to generate cyclopropanated derivatives that are very rare in nature but possess useful chemical and biological properties.

**Figure 9:**
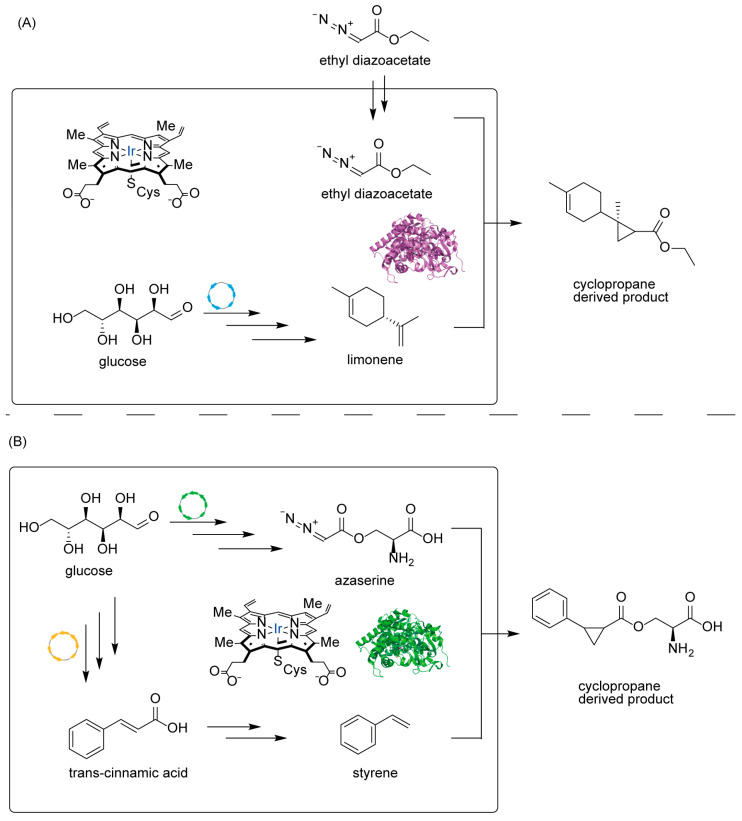
Integrated system for terpene cyclopropanation: (**A**) Integration of microbial limonene biosynthesis with iridium-catalyzed carbene transfer for cyclopropanation. (**B**) Biosynthesis of azaserine and styrene precursors for the generation of cyclopropane-containing products. The blue “Ir” indicates the iridium atom at the metal center of the artificial metalloenzyme.

The implementation of this experiment presented a microbial platform that enables de novo biosynthesis of a diazo ester carbene precursor and its application in intracellular unnatural carbene-transfer reactions. Expression of a biosynthetic gene cluster in Streptomyces albus resulted in the production of azaserine [83], an α-diazoester that served as an intracellular carbene donor. This metabolically generated reagent was employed for cyclopropanation of styrene, which was also produced intracellularly (Figure 9). The reaction was catalyzed by engineered P450 mutants utilizing native cofactors, achieving excellent diastereoselectivity and moderate yield [84]. This work establishes a foundation for integrating abiotic carbene chemistry into microbial biosynthesis pathways. Another example was the integration of artificial metalloenzymes (ArMs) that catalyze the cyclopropanation reaction with ethyl diazoacetate and limonene. Limonene was synthesized by introducing a heterologous biosynthetic pathway with phenylalanine and cinnamic acid as the key intermediates, while ethyl diazo acetate was added as a substrate [85,86]. By utilizing a transport system for iridium-based artificial cofactor, good levels of diastereoselectivity and acceptable yield were achieved, further highlighting the capability of synthetic biology and synthetic chemistry to produce molecules that were previously inaccessible to nature [87].

This accomplishment is important in the bigger picture, not just the products made. This is the first work to clarify a transformative approach to synthetic biology, combining the best of biological and chemical synthesis. The intricate biosynthetic pathways of this microbial host deliver complex terpenes, which would be difficult to achieve chemically. The enzyme, fashioned from a natural protein, introduces a non-native functionality using two different metalloenzymes. This chemo-enzymatic method utilizes biological capabilities to design sophisticated molecular architectures and the flexibility of synthetic chemistry to develop a wide spectrum of chemical transformations. The success of this hybrid method has opened the doors toward more non-natural reactions in biological systems. Thus, this may lead to much broader chemical space accessible through microbial fermentation. This integrated method could also allow for the generation of terpene derivatives with tailored properties for pharmaceutical, agricultural, and materials science applications. The research suggests how synthetic chemistry and synthetic biology can be synergistically combined for the generation of functionalities that cannot be achieved by either of the two on their own.

## 5. Applications and Case Studies of Engineered Terpenes

### 5.1. Advanced Biofuels and Bulk Chemicals from Short-Chain Terpenes

To find sustainable alternatives to petroleum-based fuels, extensive studies are being undertaken on terpene-based biofuels. Significant emphasis is being laid upon short-chain terpenes for combustion applications as they can exhibit good physicochemical properties. The compound bisabolene is a sesquiterpene that is being considered as a candidate for diesel replacement because its energy density is comparable to that of diesel, and it combusts with favorable characteristics. Engineered approaches include optimization of various mevalonate (MVA) and methylerythritol phosphate (MEP) pathways in *E. coli* and *S. cerevisiae* for high-level production. Strategies involving the fusion of enzymes have been successful in reducing the toxicity of intermediates and increasing the carbon flux to bisabolene biosynthesis [88].

The monoterpene pinene is being studied as a potential jet fuel substitute because of its high energy content and ability to be upgraded catalytically into jet fuel precursors. The toxicity of monoterpenes to their microbial host means that production efforts have focused on implementation of in situ product removal techniques such as two-phase extraction and gas stripping systems to alleviate cellular stress and improve production.

In addition to these specific compounds, terpenes as a class may offer further advantages as renewable biofuels, including lower net carbon emissions, compatibility with existing fuel infrastructure, and potential production from non-food lignocellulosic biomass. The economic viability of terpene-based biofuels is enhanced by metabolic engineering advances that have substantially raised titers and productivities, favoring their eventual commercial use as sustainable alternatives to fossil fuels in transportation.

### 5.2. Pharmaceutical and Nutraceutical Applications

The pharmaceutical industry is one of the most successful application domains for engineered terpenes. Several key accomplishments have illustrated the clinical and commercial potential of microbial terpene production. Artemisinin is a potent antimalarial sesquiterpene lactone and an important case of metabolic engineering. Microbial platforms for artemisinic acid—the precursor of artemisinin—end the supply chain limits of sourcing this from *Artemisia annua* [89,90,91,92]. To achieve this breakthrough, not only was high-level production of the 3-times oxidized amorpha-4,11-diene precursor needed, but also the functional expression of a cytochrome P450 enzyme to create artemisinic acid. The artemisinic acid was chosen as it represents one of the most complex pathway engineering accomplishments to date. The commercialization of this technology has greatly improved global access to antimalarial therapies and demonstrated that microbial systems may safely build complex plant-derived therapeutics.

Engineered microbes have also been used to produce taxadiene, the committed precursors of the anticancer drug paclitaxel [93,94]. Nevertheless, the next oxidation steps in the full paclitaxel biosynthetic pathway are still challenging due to the complexity of the P450-mediated reactions. Besides these pharmaceutical applications, engineered microbial hosts have also effectively produced nutraceutical terpenes. Nootkatone is a high-value sesquiterpene naturally occurring in grapefruit. Heterologous expression of the valencene synthase and specific cytochrome P450s that catalyze targeted oxidation reactions has enabled its synthesis in yeast and bacterial systems. The use of this compound both as a flavor and as a bug repellent is extensive. The utilization of microbial production for this is more sustainable and reliable than plant extraction. The successful microbial synthesis of these high-value terpenes demonstrates how advanced the existing metabolic engineering approaches have become. Moreover, this work provides strategies to develop production platforms of other terpenoids that are therapeutically or nutritionally relevant.

### 5.3. Frontier Applications of Non-Natural Terpenes

The development of hybrid chemo-enzymatic methods for terpene functionalization has opened new avenues in terpenoid research to access non-natural compounds with potentially new properties. By incorporating non-biological functional groups, such as cyclopropane rings, aziridines, and other synthetic moieties, terpenes can be designed to yield derivatives that may possess higher bioactivity, enhanced stability, or new properties that are absent in natural terpenes. Cycolpropane-containing terpenes created via the novel carbene transfer chemistry established by Hartwig and Keasling may exhibit modified biological activity owing to the strain and distinctive electronic features of the cyclopropane cycle. This change in structure is beneficial in improving binding tightness to biological objects or metabolic stability, which can help in obtaining competent pharmaceutical agents. The addition of nitrogen functionalities such as aziridines may expand the range of biological activities, leading to compounds with unique mechanisms for therapeutic targets.

Non-natural terpenes can be developed as advanced materials with well-defined properties beyond pharmaceuticals. Incorporating synthetic functional groups can change physical properties like hydrophobicity, glass transition temperature, and self-assembly behavior, allowing for the development of specialty polymers, lubricants, and surface coatings, among other applications. Enzymatic catalysis, even with engineered artificial enzymes, can achieve a high regioselectivity and stereoselectivity, giving rise to a structural control that is hard to reach by classical synthetic chemistry and particularly for complex terpene scaffolds. With this degree of control, terpene-based materials can be designed with tailored functional properties. In addition, non-natural terpenes could act as versatile intermediates in synthetic chemistry, containing chiral building blocks with complex functionalities that can be altered by synthetic chemistry.

This vast, unexplored chemical space can be explored further as a potential source of terpenes for a number of high-value applications in future research. As methods for making non-natural terpenes are changing, especially with the development of artificial metalloenzymes along with other hybrid biocatalysts, the diversity of accessible products and potential applications will grow rapidly, enabling innovative applications in many industrial sectors.

## 6. Conclusions and Future Perspectives

Microbial terpene production has gone from simple pathway reconstruction to engineering production systems for industrial-scale production. Scientists have found ways of improving chassis organisms, central metabolism, and biosynthetic pathways. This has led to the development of microbes that produce terpenes at a commercial scale and under competitive conditions. The strong establishment of *E. coli* and *S. cerevisiae* as powerful production hosts has provided complementary terpene biosynthetic platforms. Good commercial examples are engineered yeast for artemisinin.

**Figure 10:**
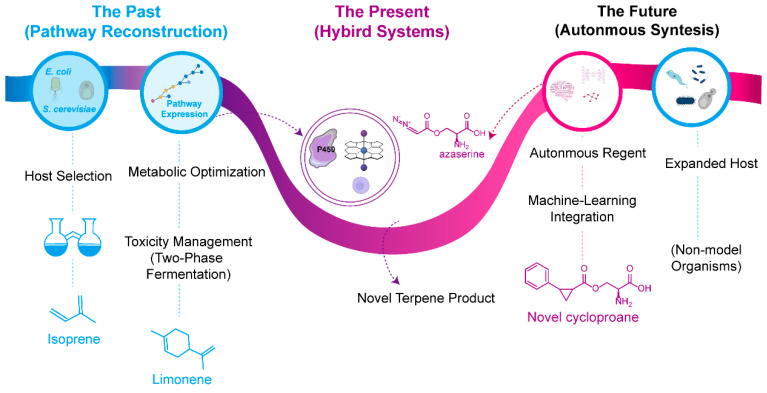
The evolution of microbial terpene production. The development of terpene biosynthesis has progressed through three major stages. The past has focused on pathway reconstruction, beginning with host selection—such as *E. coli* and *S. cerevisiae*—and metabolic optimization to establish functional expression of the mevalonate (MVA) and methylerythritol phosphate (MEP) pathways. Strategies including toxicity mitigation, such as two-phase fermentation, have enabled the production of early targets like isoprene and limonene. The present era emphasizes hybrid systems that integrate synthetic biology with abiotic chemical processes, exemplified by engineered cytochrome P450 enzymes catalyzing carbene transfer reactions to generate terpene scaffolds beyond those found in nature. The future envisions autonomous biosynthetic platforms, where machine learning-guided design, expanded utilization of diverse microbial hosts—including non-model organisms—and integrated biocatalytic and chemocatalytic processes will enable systematic exploration of unprecedented terpene chemical space.

The next stage is to do more than optimize the repertoire we have (Figure 10). We also need to create new pathways and capabilities beyond natural biosynthesis. The introduction of abiological chemistry will change the paradigm of metabolic engineering from copying nature to expanding its functional frontier. Through pioneering work on carbene transfer chemistry, we have facilitated the integration of biological synthesis with synthetic chemistry. Furthermore, microbes are capable of producing complex terpene scaffolds, while engineered enzymes can achieve interesting transformations not found in nature.

Future research directions will focus on several key areas. The creation of new artificial enzymes that can speed up other non-natural reactions, such as C-H amination and olefin metathesis, will greatly enhance the synthetic capacity of living cells. The creation of microbial systems that can produce abiotic materials like diazo compounds will make fully autonomous, self-sufficient systems feasible. Machine learning and artificial intelligence-based methods will assist with the identification of optimal pathways and direct protein engineering approaches for enzyme design having novel catalytic functions. Additionally, a more extensive range of existing model organisms, non-native microbial hosts such as cyanobacteria for production via photosynthesis, and thermophiles for better product recovery will yield more probable production platforms.

The application of these advances will give rise to microbial systems functioning as programmable chemical factories, which can produce natural terpenes as well as rationally designed molecules having specific and tailored structural features. This ability can be used for pharmaceuticals, material science, and to make sustainable alternatives to chemicals from petroleum. The future of making terpenes in microbes is to develop new functions that take biological chemistry beyond its natural limits and enable the merging of biological and chemical synthesis.
